# Fabrication and Performance Enhancement of Wood Liquefaction-Based Carbon Fibers Modified with Alumina Nanoparticles

**DOI:** 10.3390/polym17020155

**Published:** 2025-01-09

**Authors:** Linshuang Gan, Yijing Liu, Zaibirinisa Yimin, Jianglong Wu, Jialin Lv, Zhigao Liu

**Affiliations:** 1 College of Resources, Environment and Materials, Guangxi University, Nanning 530004, China; gls2237@163.com (L.G.); 15097908220@163.com (Y.L.); 13095165819@163.com (Z.Y.); 15086272032@163.com (J.W.); lvjialin1998@163.com (J.L.); 2 State Key Laboratory of Featured Metal Materials and Life-Cycle Safety for Composite Structures, Guangxi University, Nanning 530004, China

**Keywords:** aluminum oxide nanoparticles, carbon fibers from wood liquefiers, heat resistance, tensile strength

## Abstract

In this paper, alumina-modified wood liquefaction (AL-WP) was prepared by blending nano-alumina (Al_2_O_3_) into wood liquefaction phenolic resin (WP) using a co-blending method. Alumina-modified wood liquefaction protofilament fiber (AL-WPF) was obtained by melt-spinning, curing, and thermo-curing processes, which were followed by carbonization to obtain alumina-modified wood liquefaction carbon fiber (AL-WCF). This paper focuses on the enhancement effect of nano-alumina doping on the mechanical properties and heat resistance of wood liquefaction carbon fiber (WCF), explores the evolution of graphite microcrystalline structure during the high-temperature carbonization process, and optimizes the curing conditions of AL-WPF. The results showed that the introduction of Al_2_O_3_ significantly improved the mechanical properties and heat resistance of carbon fibers. When 1.5% Al_2_O_3_ was doped and carbonized at 1000 °C, the tensile strength of AL-WCF was increased from 33.78 MPa to 95.74 MPa, there was an enhancement of 183%, its residual carbon rate could reach 79.2%, which was better than that of the undoped wood liquefaction (WCF), and it exhibited a more substantial heat-resistant property. In addition, the best curing process for alumina nanoparticle wood liquefiers was obtained by optimizing the curing conditions: hydrochloric acid concentration of 16%, formaldehyde concentration of 18.5%, temperature increase rate of 15 °C/min, holding time of 3 h, and holding temperature of 100 °C. These studies provide a theoretical basis and technical support for developing and applying carbon fibers from alumina-modified wood liquefiers.

## 1. Introduction

Carbon fiber, as an inorganic polymer fiber material with excellent properties, has a high carbon content, which gives it a promising application in a wide range of industrial fields, including chemical engineering, precision machinery construction, structural building engineering, and even nuclear energy facilities as well as many other high-end technologies [1]. As carbon fibers are constrained by non-renewable raw materials, complex processes, and high production costs, scholars worldwide have focused on exploring cost-effective and high-performance carbon fiber modification methods [2,3]. In recent years, natural biomass resources such as bamboo [4,5], wood [6] and hemp plants [7] have begun to be widely used in the development and manufacture of various carbon-based functional and composite materials.

Current research on carbon fiber modification primarily focuses on enhancing mechanical strength, improving thermal stability, optimizing electrical properties, and reinforcing composite strength [8,9,10,11]. Among these efforts, reducing structural defects (e.g., skin–core structure, cracks, and voids) in carbon fibers is crucial for the overall improvement of their properties [12]. As an emerging material, carbon fiber from wood liquefiers has attracted much attention due to its renewable raw material, low cost, and simple production process. However, its development still faces challenges, the most notable of which is the presence of structural defects, especially the skin–core structure [13,14]. The precursor of carbon fiber from wood liquefaction is a thermoplastic biomass-based resin with many aromatic ring structures, which is spinnable but fragile and, therefore, needs to be cured to form a three-dimensional cross-linked structure [15,16,17]. However, the highly reactive counterparts in the resin molecules will promote rapid cross-linking and densification of the surface of the fiber during the curing process, thus preventing the cross-linking small molecules from entering the interior of the fiber and making the core layer cross-linking incomplete, which results in the production of a skin–core structure of the cured carbon fiber protofilaments, which in turn affects the mechanical and heat resistance of the carbon fiber [18,19]. Zhang et al. treated the raw filaments of wood liquefiers with boric acid impregnation to improve their mechanical properties. However, since the carbon fibers from wood liquefiers formed a skin–core structure during the curing process of the raw filaments, the modification method had a limited effect on the structure of the carbon fibers, and the enhancement of the mechanical strength was not significant [20]. Meanwhile, to enhance the mechanical properties of phenolic-based carbon fibers, Yun et al. employed a blending method to incorporate alumina nanoparticles into a thermoplastic phenolic resin. During the curing process, the alumina nanoparticles reacted with hydrochloric acid in the curing solution, facilitating the rapid diffusion of +CH_2_OH carbocations into the fiber interior. This process resulted in a more homogeneous cross-linking of the phenolic fibers. As a result of the modification, the residual carbon content of the carbon fibers increased by 13.6%, and the tensile strength reached 201 MPa [21]. Similarly, Liu et al. prepared boron-modified wood liquefaction carbon fibers by reacting boric acid with the precursor, which effectively improved the mechanical properties of the carbon fibers, with the tensile strength and elongation at break increased by 117% and 86%, respectively, up to 331.57 MPa and 7.57% [22]. In summary, recent advances in carbon fiber modification have emphasized addressing structural defects, such as the skin–core structure, to enhance mechanical, thermal, and electrical properties, with notable progress in utilizing renewable wood liquefiers and chemical treatments to improve the performance of biomass-based carbon fibers.

Compared to other types of carbon fibers with well-established processing technologies, wood liquefier-based carbon fibers still have significant potential for improvement in terms of structural homogenization, mechanical properties, and thermal performance. As a result, current research primarily focuses on using wood liquefier fibers for the production of activated carbon fibers with relatively few studies dedicated to the performance modification of wood liquefier-based carbon fibers [23,24,25]. Nano-alumina, known for its excellent heat resistance, outstanding mechanical properties, and low manufacturing cost, has been widely utilized in the modification of carbon fibers and their composites. However, no relevant studies have been reported on its application to carbon fibers derived from wood liquefiers [26,27,28]. There remains an urgent need for an in-depth and systematic investigation into the mechanisms by which alumina nanoparticles influence the mechanical and thermal properties of carbon fibers from wood liquefiers, offering new directions and significant potential for research in this field.

In this study, nano-alumina was incorporated into wood liquefaction precursors using a blending method, and nano-alumina-modified wood liquefaction carbon fibers were prepared through spinning, curing, and carbonization processes. The effects of alumina nanoparticles on the structure, mechanical properties, and thermal resistance of wood liquefaction carbon fibers were systematically investigated, and the production process parameters were optimized. This work provides valuable reference data and practical significance for further research and applications of wood liquefaction carbon fibers and their composites.

## 2. Materials and Methods

### 2.1. Materials

Fir (*Cunninghamia lanceolata* (Lamb.) Hook.) was obtained from Qiqun Wood Industry Co. (Chongqing, China) and processed into 80-mesh wood powder. Phenol was supplied by Chongqing Chuandong Chemical Co., Ltd. (Chongqing, China), while nano-alumina (60 nm) was purchased from Suzhou Yuante New Material Technology Co., Ltd (Suzhou, China). Concentrated hydrochloric acid (37%), concentrated nitric acid (68%), and formaldehyde solution (37%) were provided by Chengdu Keroni Chemical Co (Chengdu, China). All the above reagents were analytically pure and can be used directly without any treatment.

### 2.2. Preparation of WP

Fir wood powder was added to a three-necked flask with phenol (mass ratio 1:6) and concentrated sulfuric acid (5% of the mass of phenol). After installing a condensation reflux device, it was heated to 160 °C and reacted for 2 h in an oil bath. At the end of the reaction, it was cooled and filtered, and the liquefaction product was collected and set aside. Subsequently, 10 g of wood liquefaction was taken and added with hexamethylenetetramine, equivalent to 5% of the mass of the liquefaction, and heated to 120 °C at a temperature increase rate of 2 °C/min. The reaction was kept at this temperature with continuous stirring for 30 min to obtain wood liquefaction phenolic resin (WP).

### 2.3. Preparation of Aluminum Oxide-Modified WPFs

The initial fibers were prepared using a melt-spinning method. Resin (2–4 g) was combined with varying mass fractions of alumina nanoparticles (0.5%, 1%, 1.5%, 2%, and 2.5% of the resin mass). The mixture was added to the heated reaction tube of the melt spinning machine. The temperature was raised to 120 °C and maintained for 30 min, after which nitrogen gas was introduced to apply appropriate pressure, ensuring the spinning solution flowed uniformly from the spinneret. The initial fibers, including wood liquefaction protofilament fibers (WPFs) and alumina-modified wood liquefaction protofilament fibers (AL-WPFs), were collected using winding rollers rotating at a speed of 80 r/min. Subsequently, the initial fibers were fully immersed in a curing solution composed of hydrochloric acid (14% concentration) and formaldehyde (18.5% concentration). The fibers were placed in a water bath and heated at a rate of 15 °C/h until the temperature reached 90 °C. They were maintained at this temperature for 2 h, then removed and rinsed thoroughly with deionized water. Finally, the fibers were placed in an oven at 160 °C for 2 h for heat curing treatment.

### 2.4. Optimal Curing Conditions for the Preparation of WPFs

The optimum addition of alumina nanoparticles was determined to be 1.5%, which was based on the mechanical property testing of the protofilaments during the heat curing stage. The prepared initial fibers with 1.5% nano-alumina were immersed in mixed solutions of hydrochloric acid (with concentrations of 12%, 14%, and 16%) and formaldehyde (concentration of 18.5%). The fibers were then heated to specified temperatures (80 °C, 90 °C, and 100 °C) at a controlled heating rate of 15 °C/min and held at these target temperatures for durations of 1 h, 2 h, and 3 h to complete the curing process. Upon completion, the fibers were removed, washed thoroughly with deionized water, and dried to obtain the cured protofilaments. The optimum process conditions were ultimately identified through thermogravimetric analysis (TG).

### 2.5. Preparation of WCFs

The prepared WPFs and AL-WPFs (with a nano-alumina content of 1.5%) were placed in alumina boats and inserted into the heating zone of a tube furnace. After sealing the flanges, nitrogen gas was introduced, and the equipment’s airtightness was verified. The temperature was uniformly increased from 30 °C to the target carbonization temperatures (800 °C, 1000 °C, 1200 °C, and 1400 °C) at a heating rate of 4 °C/min and maintained at each target temperature for 2 h. After the carbonization process was completed, the system was allowed to cool to room temperature. The carbon fibers were then removed, thoroughly washed with deionized water, and dried in a forced-air drying oven at 80 °C to obtain wood liquefaction carbon fiber (WCF) and nano-alumina-modified wood liquefaction carbon fiber (AL-WCF).

### 2.6. Characterization

The chemical composition and structural changes during the transformation from AL-WP to AL-WPF were analyzed using an infrared spectrometer (IRTracer-100, Kyoto, Japan). The thermogravimetric behavior of the samples was investigated using a differential thermogravimetric analyzer (DTG-60(H), Kyoto, Japan). The morphology and microstructure of the samples were characterized using a scanning electron microscope (S-3400N, Tokyo, Japan). The crystalline structure and degree of graphitization were examined using an X-ray diffractometer (Rigaku D/MAX 2500x, Kyoto, Japan) and a reflective laser Raman spectrometer (in-Via, Landon, UK). The tensile strength and elongation at break of liquefied wood protofilaments and carbon fibers carbonized at different temperatures were tested using a short fiber mechanical performance tester (JSF08, Shanghai, China), both before and after alumina modification, to comprehensively assess the impact of alumina modification on their mechanical properties.

## 3. Results and Discussion

### 3.1. Surface Morphology of WP

**Figure 1:**
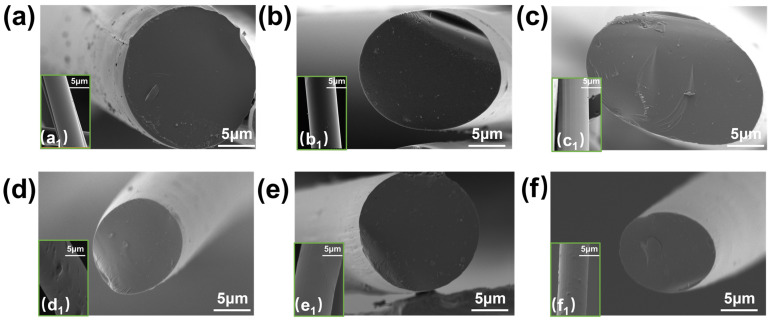
SEM images of initial fibers with different alumina additions: (**a**) cross-sectional surface of WP, (**a_1_**) radial surface of WP; (**b**) cross-sectional surface of 0.5% AL-WP, (**b_1_**) radial surface of 0.5% AL-WP; (**c**) cross-sectional surface of 1% AL-WP, (**c_1_**) radial surface of 1% AL-WP; (**d**) cross-sectional surface of 1.5% AL-WP, (**d_1_**) radial surface of 1.5% AL-WP; (**e**) cross-sectional surface of 2% AL-WP, (**e_1_**) radial surface of 2% AL-WP; (**f**) cross-sectional surface of 2.5% AL-WP, (**f_1_**) radial surface of 2.5% AL-WP.

As observed in Figure 1, panels a–f show the cross-sectional surfaces of WP and AL-WP, while panels a_1_–f_1_ display the radial surfaces of WP and AL-WP. The cross-sectional surface of WP exhibits no noticeable grooves or defects, and the radial surface is smooth and dense, indicating a continuous and ordered structure formed by the spinning solution (Figure 1a). With increasing nano-alumina content, defects in the form of particle aggregates begin to appear on the surface of the initial fibers, and the structural order decreases. As shown in Figure 1d_1_–f_1_, as the amount of nano-alumina increases, the defects on the surface of the initial fibers become more pronounced with an increase in particles and a rougher surface. This may be attributed to the addition of nano-alumina, which leads to a decrease in the uniformity of the internal structure of the initial fibers. Furthermore, the cross-sectional observation reveals that all carbon fibers exhibit a dense structure with no apparent voids.

### 3.2. Chemical Structure of WP

**Figure 2:**
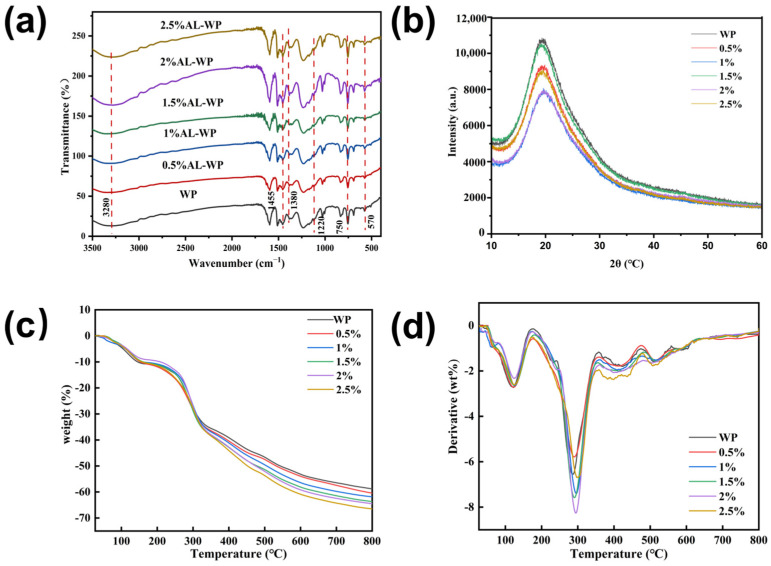
(**a**) Infrared spectra of WP and AL-WP; (**b**) XRD patterns of WP and AL-WP; (**c**) TG curves of WP and AL-WP; (**d**) DTG curves of WP and AL-WP.

Figure 2a compares the infrared spectra of WPF and its nanocrystalline alumina-modified version. The absorption peak at 1593 cm^−1^ is attributed to the stretching vibration of the C=C bond in the aromatic ring, while the stretching vibration of alkyl C-H bonds is observed in the 3000–2800 cm^−1^ region. The absorption peaks at 1593 cm^−1^, 1509 cm^−1^, and 1455 cm^−1^ correspond to the skeletal vibrations of the benzene ring, while those at 1455 cm^−1^ and 1381 cm^−1^ are attributed to the bending vibrations of alkyl C-H bonds. The peak at 1231 cm^−1^ is associated with the C-O stretching vibration of phenols, and the peaks at 1170 cm^−1^ and 1120 cm^−1^ correspond to the in-plane bending vibrations of C-H bonds in the aromatic ring. The peak at 1027 cm^−1^ corresponds to the C-O-C stretching vibration of ether bonds, while the peaks at 832 cm^−1^, 754 cm^−1^, and 690 cm^−1^ are attributed to the out-of-plane bending vibrations of C-H bonds in the benzene ring [29,30]. Overall, incorporating nanocrystalline alumina does not induce significant structural changes in the WPF, which is likely due to the lack of chemical reaction between the alumina and the polymer matrix.

Figure 2b presents the X-ray diffraction (XRD) patterns of WP and AL-WP. As shown in the figure, both WP and AL-WP exhibit the strongest diffraction peak at approximately 2θ ≈ 20°, corresponding to the (002) plane of graphite crystallites. The diffraction peak at the (002) plane is most pronounced for WP, showing a higher peak intensity. With the increasing content of nanocrystalline alumina, the intensity of the diffraction peak gradually decreases, and the peak broadens. At this stage, no significant difference is observed between the diffraction peaks of AL-WP and WP.

Figure 2c,d present the TG and DTG results for WP and AL-WP with varying amounts of nanocrystalline alumina. The TG and DTG curves of WP and AL-WP exhibit similar trends. The residual carbon content of WP is 41.18%, whereas when the nanocrystalline alumina content is 0.5%, AL-WP the residual carbon is 39.56%, indicating that WP has a slightly higher residual carbon than AL-WP. With the increase in nanocrystalline alumina content, the thermal stability of AL-WP decreases, which is possibly due to the aggregation of alumina nanoparticles in the resin and insufficient reaction during the initial stage of fiber formation [31]. During the heating process, mass loss occurs due to the release of moisture and decomposition products from lignin (such as CH_3_, CO, HCHO, and CO_2_) [32]. The mass loss process is mainly divided into three stages: the first stage (40–170 °C) involves the evaporation of bound water and the volatilization of organic compounds; the second stage (170–330 °C) is primarily due to the significant loss of lignin side chains. The addition of nanocrystalline alumina leads to a slightly higher mass loss in the initial fibers than in the control group, which is possibly due to more side chains. After 600 °C, the degradation of lignin units is mainly complete, and the TG curve becomes stable. The residual carbon of AL-WP slightly decreases, which is speculated to be related to the aggregation of alumina nanoparticles in the resin and their insufficient reaction during the initial fiber formation stage.

### 3.3. Structure and Characterization of WPF and AL-WPF

**Figure 3:**
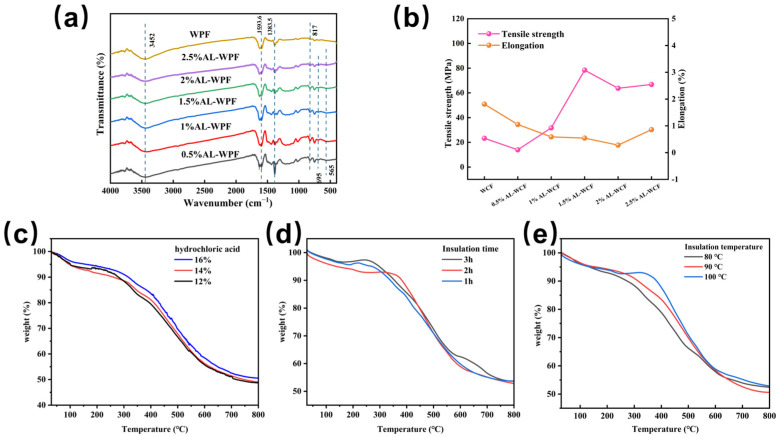
(**a**) Infrared spectra of WPF and AL-WPF under thermal curing conditions; (**b**) tensile strength and elongation at break of WPF and AL-WPF under thermal curing conditions; (**c**) TG curves of AL-WPF under curing conditions with different hydrochloric acid concentrations; (**d**) TG curves of AL-WPF under curing conditions with different holding times; (**e**) TG curves of AL-WPF under curing conditions with different holding temperatures.

The effect of different amounts of nanocrystalline alumina on the chemical composition of WPF during the curing stage is shown in Figure 3a. The absorption peak at 3452 cm^−1^ corresponds to the stretching vibration of the phenolic O-H group. With the addition of nanocrystalline alumina, this peak shifts, indicating increased hydrogen bonding strength within the WPF. The absorption peak at 1383 cm^−1^ is attributed to the bending vibration of alkyl C-H bonds [33]. During the curing reaction, the phenolic hydroxyl groups undergo condensation to form -CH₂-O-CH_2_- linkages. As the curing time increases, the reaction between -CH_2_OH groups in the curing resin and the aromatic rings in the fiber molecules intensifies, resulting in an increased number of cross-linking groups, such as methylene bridges (-CH_2_-) and ether linkages (-CH_2_-O-CH_2_-). At this stage, the intensity of the characteristic absorption peak at 1480 cm^−1^, corresponding to the deformation vibration of -CH_2_- between aromatic rings, increases, while the peaks at 748 cm^−1^ and 817 cm^−1^ become relatively weaker. The absorption peaks at 695 cm^−1^ and 565 cm^−1^ are attributed to the bending vibrations of Al-O bonds [34], indicating that nanocrystalline alumina is present in the form of Al-O bonds within the raw fibers during the curing stage.

To investigate the effect of nanocrystalline alumina content on the mechanical properties of WPF, WPF samples with varying amounts of nanocrystalline alumina were prepared under identical curing and heat-curing conditions. Their tensile strength and elongation at break were then measured. The results are shown in Figure 3b. Under curing conditions with a hydrochloric acid concentration of 14% and a heat-curing temperature of 160 °C, the tensile strength and elongation at break of WPF were 23.26 MPa and 1.81%, respectively. As the tensile strength of AL-WPF increased, the elongation at break gradually decreased. Notably, when the nanocrystalline alumina content was 1.5%, the average tensile strength of AL-WPF increased to 78.40 MPa, while the average elongation at break decreased to 0.54%. It is speculated that the Al-O bonds present in AL-WPF may have enhanced the toughness of the fibers, thereby improving the mechanical properties of WPF. However, when the nano-Al_2_O_3_ content exceeded 1.5%, the tensile strength decreased. It is speculated that this is due to the increased nano-Al_2_O_3_ content consuming the hydrochloric acid in the curing solution, which leads to a decrease in acid concentration. Additionally, the formation of pores on the surface and within the fibers may reduce the fiber strength [35]. In conclusion, the optimal nano-alumina content is 1.5%, as it enhances the mechanical properties of WPF to some extent.

The effect of different hydrochloric acid concentrations, holding temperatures, and holding times on the thermal stability of AL-WPF was investigated using 1.5% AL-WPF as a base, as shown in Figure 3c–e. Figure 3c shows that the carbon residue reached its highest value of 50.5% when the hydrochloric acid concentration in the curing solution was 16%. As the hydrochloric acid concentration decreased, the carbon residue decreased, with a value of 48.72% at a concentration of 12%. It is speculated that the phenolic hydroxyl groups in the curing solution activate the ortho- and para-positions of the phenolic ring. When these positions are attacked by the +CH_2_OH carbocation, ortho- or para-hydroxy methyl phenols are formed. These hydroxy methyl phenols then react with phenol or other hydroxy methyl phenols, forming a cross-linked network structure, which enhances stability at high temperatures. The higher the concentration of hydrochloric acid, the greater the amount of +CH_2_OH generated in the solution, resulting in a higher degree of cross-linking of the fibers, thereby improving their mechanical properties and thermal stability. The TG curves for different holding times under the conditions of 16% hydrochloric acid concentration, an initial temperature of 30 °C, a curing temperature of 90 °C, and a heating rate of 15 °C/min are shown in Figure 3d. When the holding time is 3 h, the carbon residue reaches its maximum value of 53.71%. As the curing time is extended, the curing duration of the fibers at high temperatures also increases, allowing the +CH₂OH groups to penetrate fully into the core layer of the fibers, promoting cross-linking both inside and outside the fibers. The fibers are connected by methylene ether bonds and methylene bridges, with the methylene bridges exhibiting higher stability, thereby enhancing the overall stability of the fibers [36]. The TG curves for different holding times under the conditions of 16% hydrochloric acid concentration, an initial temperature of 30 °C, a heating rate of 15 °C/min, and a holding time of 3 h are shown in Figure 3e. When the holding temperature is 100 °C, the carbon residue reaches its maximum value of 53.15%, which is considered the optimal holding temperature. In conclusion, the optimal process conditions for the precursor fibers are a 16% hydrochloric acid concentration, an 18.5% formaldehyde concentration, a 15 °C/min heating rate, a holding time of 3 h, and a holding temperature of 100 °C.

### 3.4. Structure and Characterization of Nano-Alumina-Modified WCFs

**Figure 4:**
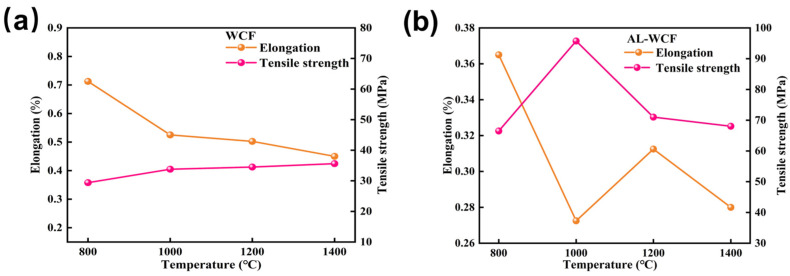
Tensile strength and elongation at break of nano-alumina-modified wood liquefaction carbon fibers at different temperatures: (**a**) WCF; (**b**) AL-WCF.

Figure 4a,b illustrate the effect of different carbonization temperatures on the mechanical properties of WCF and AL-WCF. The tensile strength was improved to some extent under different carbonization temperatures, particularly at the carbonization condition of 1000 °C. The tensile strength and elongation at the break of WCF were 33.78 MPa and 0.52%, respectively. After adding 1.5% nanocrystalline alumina, the tensile strength increased to 95.74 MPa, while the elongation at break decreased to 0.27%. The tensile strength of the modified wood liquefied fiber significantly increased, further demonstrating that nanocrystalline alumina modification effectively improves the mechanical properties of carbon fibers and enhances their overall mechanical performance to some extent. However, as the carbonization temperature exceeds 1000 °C, the tensile strength of AL-WCF gradually decreases. It is speculated that this is due to the increase in surface porosity of the carbon fibers with higher temperatures, which leads to a reduction in packing density, thereby affecting their mechanical properties [37]. Under the carbonization condition of 1000 °C, the tensile strength of WCF gradually increases, but it remains lower than that of the modified AL-WCF. Overall, the mechanical properties of the nano-alumina-modified wood liquefaction carbon fibers are optimal at a carbonization temperature of 1000 °C. Therefore, nano-alumina can effectively improve the tensile strength of carbon fibers to some extent.

A one-way analysis of variance (ANOVA) was conducted to evaluate the effect of different carbonization temperatures on the tensile strength of AL-WCF, as shown in Table 1, F(3,8) = 21.369, with a *p*-value < 0.01. Since the *p*-value is smaller than the significance level (α = 0.05), the null hypothesis is rejected, indicating that there is a significant difference in the tensile strength of AL-WCF under different carbonization temperatures.

**Figure 5:**
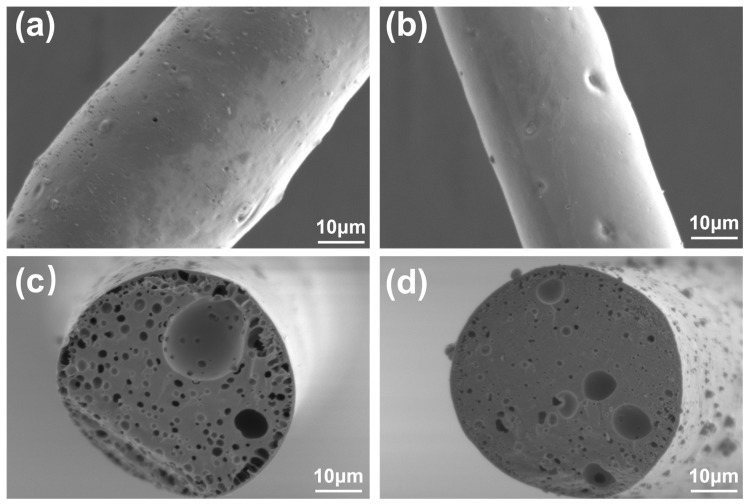
The SEM images of carbon fibers at a carbonization temperature of 1000 °C: (**a**) represents the radial surface of WCF; (**b**) represents the radial surface of AL-WCF; (**c**) shows the cross-sectional surface of WCF; (**d**) shows the cross-sectional surface of AL-WCF.

Figure 5 shows the SEM images of carbon fibers at a carbonization temperature of 1000 °C. Figure 5a,c show that the surface of WCF exhibits a distinct porous structure with the edges of the cross-section being relatively rough and the skin–core structure more pronounced. Additionally, the surface and core layer of the carbon fibers display uneven graphitization, leading to an increased disparity between the external and internal structures. After nano-alumina modification, the carbon fibers shown in Figure 5b,d exhibit a more orderly and uniformly compact cross-sectional structure. The skin–core structure is less pronounced than the unmodified fibers, and tiny pores are observed in the central region. This is speculated to be due to the gases generated during the high-temperature decomposition reaction in the carbonization process of nano-alumina, which may have caused defects in the fiber structure.

**Figure 6:**
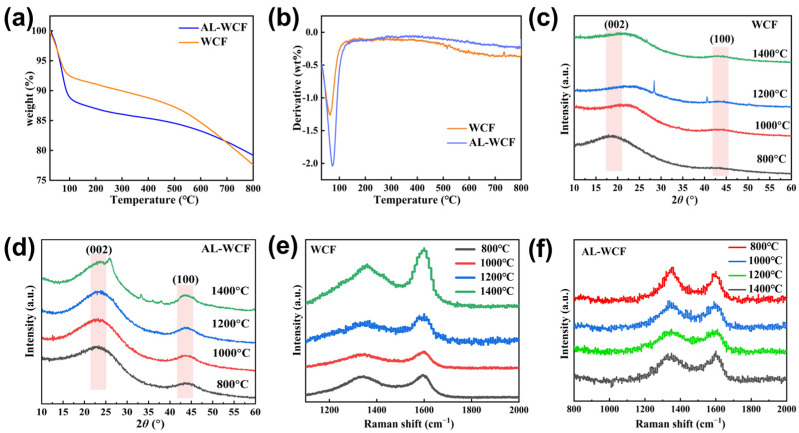
(**a**) TG curves of WCF and AL-WCF; (**b**) DTG curves of WCF and AL-WCF; (**c**) XRD spectra of WCF at different carbonization temperatures; (**d**) XRD spectra of AL-WCF at different carbonization temperatures; (**e**) Raman spectra of WCF at different carbonization temperatures; (**f**) Raman spectra of AL-WCF at different carbonization temperatures.

Thermogravimetric analysis was performed on WCF and Al-WCF prepared at the same carbonization temperature, and the thermogravimetric (TG) and differential thermogravimetric (DTG) curves are shown in Figure 6a,b. The carbon residue of WCF prepared at a carbonization temperature of 1000 °C was 77%, while the carbon residue of Al-WCF with 1.5% nano-alumina addition was 79.2%. Additionally, the weight loss rate of Al-WCF above 200 °C was significantly lower than that of WCF, indicating that nano-alumina has excellent thermal resistance and that its addition effectively improves the thermal stability of the carbon fibers.

Figure 6c,d shows the X-ray diffraction patterns of WCF and Al-WCF. As the temperature increases, the 002 diffraction peak of WCF gradually shifts to the right with the 2θ angle increasing from 19.25° to 21.77° and the graphite crystallite interlayer spacing (d_002_) gradually decreasing to 0.408 nm (Table 2). In contrast, for Al-WCF, at a carbonization temperature of 800 °C, the 002 diffraction peak reaches a 2θ angle of 23.00° (Table 3), and a distinct (100) diffraction peak appears with a graphite crystallite interlayer spacing (d_002_) of 0.385 nm [38]. The carbonization temperature has little effect on the crystal structure parameters of Al-WCF, and there is no clear trend in the 2θ angle variation at different carbonization temperatures. This suggests that compared to WCF, Al-WCF can form a more ordered and regular graphite crystallite structure at lower carbonization temperatures. However, increasing the carbonization temperature does not significantly optimize the alignment of the graphite crystallite structure within Al-WCF. Based on the analysis of the literature and SEM images, it is inferred that during the curing process of the precursor fibers, the pore structure formed in Al-WCF effectively improves the skin–core structure, enhancing the uniformity of the fibers. This results in higher carbon network polymerization at lower carbonization temperatures, leading to a more stable carbon network structure.

Figure 6e,f show the Raman spectra of WCF and AL-WCF at different carbonization temperatures. It can be observed that the Raman spectrum of WCF shows a typical saddle-shaped structure and has two prominent peaks along the fiber axis—the D peak at 1335 cm^−1^ and the G peak at 1595 cm^−1^, respectively. The D peak represents the carbon atom lattice defects, while the G peak represents the ordered graphite lattice [39]. According to the fitting results of Raman spectroscopy data (Table 4 and Table 5), the graphitization degree of AL-WCF gradually increased with the increase in carbonization temperature, which is manifested by the shift of the D peak to a higher wave number and the gradual enhancement of the intensity of the G peak. At the same time, the half-height widths of the D peak and the G peak were also expanded. These changes indicate that although high-temperature carbonization helps to enhance the graphitization degree of AL-WCF and gradually transforms its carbon fiber structure into an ordered graphitic microcrystalline structure, the crystalline defects are still not eliminated.

## 4. Conclusions

Doping nanocrystalline Al_2_O_3_ into wood-derived fibers (WPFs) can enhance the cross-linking degree of the fibers after curing to a certain extent, thereby improving their thermal stability and char residue. As the doping amount of nanocrystalline Al_2_O_3_ increases, the tensile strength of the fibers shows varying degrees of improvement. When 1.5% nanocrystalline Al_2_O_3_ was added and carbonized at 1000 °C, the slag ratio of modified wood fiber (AL-WCF) reached 79.2%, and the thermal stability of the modified wood fiber (AL-WCF) was higher than that of unincorporated wood fiber (WCF). In addition, the tensile strength increased to 95.74 MPa, which is 65.96 MPa higher than the original WCF, representing an increase of 183%. During the carbonization process, the nanocrystalline Al_2_O_3_-doped wood fibers not only exhibited improved high-temperature resistance but also showed improvements in the degree of graphitization and the core–shell structure of the fibers. This suggests that alumina modification effectively promoted the optimization of the internal structure of the fibers, thereby enhancing their overall mechanical performance and thermal stability. These findings suggest that nano-Al_2_O_3_, as a modification additive, plays a crucial role in improving the high-temperature performance and mechanical strength of wood-derived fibers, providing a novel approach for their high-performance applications.

## Figures and Tables

**Table 1:** Results of univariate variance analysis of AL-WCF tensile strength at different carbonization temperatures.

ANOVA					
Tensile Strength Values of AL-WCF					
	Sum of Square	Degree of Freedom	Mean Square	F	Significance
Inter-group	1690.605	3	563.535	21.369	0.000
Intra-group	210.971	8	26.371		
total	1901.576	11			

**Table 2:** Crystal structure parameters of WCF at different carbonization temperatures.

Temp (°C)	2*θ*_002_ (°)	2*θ*_100_ (°)	d_002_ (nm)	L_c_ (nm)	L_c_/d_002_
800	19.251	42.533	0.461	0.691	1.49
1000	20.851	42.873	0.426	0.946	2.22
1200	21.518	43.125	0.412	0.706	1.71
1400	21.770	44.311	0.408	0.666	1.63

**Table 3:** Crystal structure parameters of AL-WCF at different carbonization temperatures.

Temp (°C)	2*θ*_002_ (°)	2*θ*_100_ (°)	d_002_ (nm)	L_c_ (nm)	L_c_/d_002_
800	23.044	43.629	0.385	0.660	1.71
1000	23.207	43.540	0.383	0.652	1.70
1200	23.800	43.377	0.373	0.642	1.72
1400	25.816	43.718	0.345	0.677	1.96

**Table 4:** Raman spectral parameters of WCF at different carbonization temperatures.

Temp (°C)	W_D_ (cm^−1^)	W_G_ (cm^−1^)	FWHM (D) (cm^−1^)	FWHM (G) (cm^−1^)	I_D_	I_G_	R (I_D_/I_G_)
800	1348.30	1589.73	256.33	100.12	27,390.51	10,854.74	2.52
1000	1347.39	1592.29	267.23	92.12	18,801.96	7285.58	2.58
1200	1359.26	1594.12	292.81	92.86	28,183.44	11,197.3	2.52
1400	1363.49	1594.91	260.06	81.49	52,288.59	23,809.34	2.20

**Table 5:** Raman spectral parameters off AL-WCF at different carbonization temperatures.

Temp (°C)	W_D_ (cm^−1^)	W_G_ (cm^−1^)	FWHM (D) (cm^−1^)	FWHM (G) (cm^−1^)	I_D_	I_G_	R (I_D_/I_G_)
800	1355.49	1588.58	212.39	105.72	2798.78	1404.97	1.99
1000	1357.39	1589.82	241.60	118.26	2604.26	1266.92	2.06
1200	1352.37	1588.25	203.29	108.46	2436.60	1319.67	1.85
1400	1351.25	1591.81	155.93	98.62	2296.69	1334.78	1.72

## Data Availability

The original contributions presented in this study are included in the article. Further inquiries can be directed to the corresponding author.
